# Eficacia de la tafenoquina en la profilaxis y tratamiento de la malaria por *Plasmodium vivax*, revisión sistemática y metaanálisis

**DOI:** 10.7705/biomedica.5988

**Published:** 2022-06-01

**Authors:** Astrid Lorena Cubillos, Alexandra Porras, Alejandro Rico

**Affiliations:** 1 Departamento de Epidemiología, Facultad de Medicina, Universidad El Bosque, Bogotá, D.C., Colombia Universidad El Bosque Departamento de Epidemiología Facultad de Medicina Universidad El Bosque Bogotá, D.C. Colombia

**Keywords:** Malaria *Vivax*, tratamiento, profilaxis, tafenoquina, primaquina, *Vivax* malaria, treatment, prophylaxis, tafenoquine, primaquine

## Abstract

**Introducción.:**

La tafenoquina fue aprobada en el 2018 por la *Food and Drug Administration* de Estados Unidos y, en el 2019, por la *Therapeutic Goods Administration* en Australia. Su administración en dosis única y su mecanismo de acción en las fases aguda y latente han sido objeto de estudio para cambiar el esquema de tratamiento de la malaria por *Plasmodium vivax.*

**Objetivo.:**

Evaluar la evidencia científica disponible sobre la eficacia de la tafenoquina en la profilaxis y el tratamiento de la malaria por *P. vivax*, entre el 2009 y el 2019.

**Materiales y métodos.:**

Se establecieron los descriptores MeSH y DeCS. Se utilizó la sintaxis ((Malaria *Vivax*) AND (tafenoquine) AND (prophylaxis)) OR [(Malaria *Vivax*) AND (tafenoquine) AND (relapse)] en las siguientes bases de datos: Pubmed, The Cochrane Central Register of Controlled Clinical Trials (CENTRAL), ISIS Web of Science, Lilacs y Scopus. Los resultados obtenidos se sometieron a análisis crítico (matriz CASPE). El análisis cuantitativo se realizó utilizando la diferencia de riesgos en análisis de supervivencia (Kaplan-Meier) en los tres artículos finales.

**Resultados.:**

Se sometieron tres estudios a metaanálisis (Llanos-Cuentas, 2014; Llanos- Cuentas, 2019, y Lacerda, 2019) para evaluar la eficacia del tratamiento con tafenoquina en comparación con primaquina. Se obtuvo una diferencia de riesgo global de 0,04 (IC95% 0-0,08; p=0,07). La tafenoquina no mostró inferioridad en la eficacia del tratamiento frente al esquema de primaquina.

**Conclusión.:**

La tafenoquina es una alternativa que mejora el cumplimiento del tratamiento, lo que podría acercar a Colombia a las metas de la Estrategia Técnica Mundial contra la Malaria, 2016-2030.

## Abstract (en)

**Introduction:**

Tafenoquine was approved in 2018 by the Food and Drug Administration in the United States and in 2019 by the Therapeutic Goods Administration in Australia. Its administration in a single dose and its mechanism of action in the acute and latent phases of the disease have been studied to change the treatment regimen for *Plasmodium vivax* malaria.

**Objective:**

To evaluate the available scientific evidence of the efficacy of tafenoquine in prophylaxis and treatment between 2009 and 2019.

**Materials and methods:**

We established the MeSH and DeCS descriptors and we used the syntax ((Malaria *Vivax*) AND (tafenoquine) AND (prophylaxis)) OR [(Malaria *Vivax*) AND (tafenoquine) AND (relapse)] in the following databases: Pubmed, The Cochrane Central Register of Controlled Clinical Trials (CENTRAL), ISIS Web of Science, Lilacs, and Scopus. The results obtained were subjected to critical analysis (CASPE matrix). The quantitative analysis was performed with risk differences in survival analysis (Kaplan Meier) in the final three articles.

**Results:**

Three studies underwent meta-analysis (Llanos-Cuentas, 2014; Llanos-Cuentas, 2019, and Lacerda, 2019) to evaluate the efficacy of the treatment with tafenoquine compared to primaquine. A global risk difference of 0.04 was obtained (95% CI: 0.00-0.08; p=0.07). Tafenoquine did not show inferiority in the efficacy of treatment compared to the primaquine scheme.

**Conclusion:**

Tafenoquine is a therapeutic alternative to primaquine that improves adherence, which could bring Colombia closer to the goals of the World Technical Strategy against Malaria 2016-2030.

La malaria o paludismo es una enfermedad transmisible que constituye un grave problema de salud pública. Diversos países tienen una carga desproporcionada de la enfermedad, evidenciada en el aumento de casos. En el Informe Mundial sobre el Paludismo 2020, se estimó que hubo 229 millones de casos de malaria en todo el mundo, siendo África, el Sudeste Asiático y el Mediterráneo Oriental las regiones que más casos presentaron, con más del 50 % de la carga de la enfermedad concentrada en cinco países: Nigeria (27 %), República Democrática del Congo (12 %), Uganda (5 %), Mozambique (4 %) y Níger (4 % ) ^1^.

En el 2017, la Organización Panamericana de Salud (OPS) reportó 108 millones de personas en riesgo de malaria en Latinoamérica y, a pesar de los esfuerzos para disminuir los casos de muertes, se vio un aumento de casos aproximado del 26 % en los últimos tres años, con el 93 % en Brasil, Colombia, Guyana, Haití, Perú y Venezuela. Argentina y Paraguay iniciaron desde el 2016 el proceso ante la Organización Mundial de la Salud (OMS) para la certificación de la eliminación de la malaria en el territorio, lo que Paraguay obtuvo en el 2018 y Argentina en el 2019, ya que reportaron cero casos autóctonos a partir del 2012 y el 2011, respectivamente. En las Américas se presentó una proporción de casos de *Plasmodium vivax* de 75 % y de *Plasmodium falciparum* de 20 % ^2^^).^

En Colombia, se reportaron 61.339 casos en el 2018, con un incremento del 27,94% para el 2019 con 78.513 casos y una disminución del 1,98 % para el 2020 con 76.958 casos, y una tendencia a la disminución en las notificaciones de casos con respecto al mismo corte epidemiológico del 2019. En este último periodo de 2019, 75.816 casos fueron de malaria no complicada y 1.142 de malaria complicada. Predominó la infección por *P. vivax,* con el 49,8 % (38.288 casos), seguida por *P. falciparum,* con el 49,4 % (38.017) y, la infección mixta, con el 0,8 % (653). Los principales focos de transmisión de malaria en el país se encuentran en Chocó (27,6 %), Nariño (21,5 %), Antioquia (9,5 %), Córdoba (9,2 %) y Norte de Santander (6,4 %), departamentos que aportan el 74,2 % de los casos de malaria no complicada. El 44,7 % de la población afectada es afrocolombiana y el 20,2 % es indígena ^3^^-^^5^.

La erradicación de la malaria presenta diversas problemáticas entrelazadas entre sí: el cumplimiento del tratamiento completo después de la desaparición de los síntomas, el surgimiento de resistencia de los parásitos a los medicamentos antimaláricos y de los vectores a los insecticidas, la falta de financiamiento y el aumento de la incidencia en la población vulnerable, entre otros. Por ello, la OMS lanzó la “Estrategia técnica mundial contra la malaria, 2016-2030”, con el objetivo de reducir las tasas de mortalidad y de incidencia, eliminar la malaria en los países en los que persista la transmisión y evitar su restablecimiento en los países exentos de la enfermedad. Las metas trazadas son el acceso universal a la prevención, el diagnóstico y el tratamiento oportunos, basados en el máximo control vectorial mediante una vigilancia y seguimiento entomológico adecuados, controlando la resistencia y la transmisión residual ^6^.

El tratamiento de adultos para la malaria por *P. vivax* no complicada está establecido por la OMS y depende de la sensibilidad regional a la cloroquina. En Colombia, la sensibilidad permanece alta, por lo que en la guía para la atención integral del paciente con malaria (2010) se establece como primera y segunda líneas de cura radical un esquizonticida, la cloroquina (en dosis de 10 mg por kilogramo de peso el primer día, seguidos de 7,5 mg/kg el segundo día y 7,5 mg/kg el tercer día) en conjunto con la primaquina, que actúa en la forma latente (en dosis de 0,25 mg/kg por día durante 14 días) ^7^. A partir del 2013, organizaciones internacionales como *Medicines for Malaria Venture* iniciaron en colaboración con GlaxoSmithKline las pruebas clínicas de eficacia, tolerabilidad y seguridad de la tafenoquina que, junto con el tratamiento estándar de tres días de cloroquina, promete mejorar el cumplimiento de la profilaxis y del tratamiento radical. La tafenoquina fue aprobada por la *Food and Drug Administration* (FDA) en Estados Unidos en el 2018 y por la *Therapeutic Goods Administration* (TGA) en Australia en el 2019, pero aún está pendiente la aprobación de la OMS ^8^^,^^9^.

La tafenoquina es un medicamento que pertenece a la familia de las 8-aminoquinoleínas. Fue descubierta en 1978 por el Instituto Walter Reed Army como respuesta a la búsqueda de un medicamento que actuara en las formas aguda y latente de la infección por *P. vivax*. Aún no se conoce el mecanismo de acción exacto, pero se plantea la hipótesis de que induce oxidación espontánea por ciclos *redox*, provocando la muerte del parásito. Tiene un gran volumen de distribución, escaso aclaramiento y su metabolismo es microsómico hepático por medio de citocromo P450 2D6 (CYP2D6).

Los efectos adversos son poco frecuentes, aproximadamente, el 13 %, y predominan los síntomas gastrointestinales. Se han observado *queratopatía* en *vórtice (cornea verticillate*) sin secuelas al año, prolongación del intervalo QT y anemia hemolítica en pacientes con déficit de la glucosa 6 fosfato deshidrogenasa (G6PD). Su eficacia se ha evaluado en grandes estudios como el DETECTIVE y el GATHER, los cuales son objeto de análisis de esta revisión sistemática ^10^^-^^12^.

Como propuesta para el cumplimiento internacional de la Estrategia Técnica Mundial, de los Objetivos del Desarrollo Sostenible (2030), de la Iniciativa Regional de Eliminación de la Malaria (2014) y de las acciones intersectoriales regionales posteriores a la evaluación de los resultados del Plan Decenal de Salud Pública (2012-2021), esta revisión busca evaluar la evidencia científica disponible sobre la eficacia de la tafenoquina en la profilaxis y el tratamiento radical entre el 2009 y el 2019, para así contribuir a la disminución de la incidencia y la carga de la malaria por *P. vivax* en Colombia.

## Materiales y métodos

La revisión está registrada con el número 219715 en la base de datos PROSPERO del *National Institute for Health Research.*

La pregunta PICOT (*population, patients, people*) contempló los siguientes componentes: una población de adultos (>16 años) con diagnóstico parasitológico de malaria no complicada por *P. vivax;* intervención de profilaxis o tratamiento; comparación de la administración de las 8-aminoquinoleínas y placebo; resultado expresado como eficacia, y el periodo de 2009 a 2019. El análisis cualitativo se hizo mediante revisión sistemática y, el cuantitativo, mediante metaanálisis.

### Estrategia de búsqueda

La búsqueda electrónica se inició estableciendo los siguientes descriptores MeSH y DeCs (cuadro 1)*:* malaria *vivax*, tafenoquine, prophylaxis y relapse; se utilizó la sintaxis ((*Malaria vivax*) AND (tafenoquine) AND (prophylaxis)) OR [(*Malaria vivax*) AND (tafenoquine) AND (relapse)] en las siguientes bases de datos: Pubmed, The Cochrane Central Register Of Controlled Clinical Trials (CENTRAL), ISIS Web of Science, Lilacs y Scopus.

**Cuadro 1:** Términos MESH y DeCS

Palabra	Sinónimos	Definición
Malaria *vivax*	Vivax malaria, paludismo por *Plasmodium vivax*; paludismo, *Plasmodium vivax*	Paludismo causado por *Plasmodium vivax*. Esta forma de malaria es menos grave que la malaria *falciparum*, pero existe una mayor probabilidad de que ocurran recaídas. Los paroxismos febriles a menudo ocurren día de por medio. Año de introducción: 1992 ^13^
Tafenoquina	Succinato de tafenoquina, maleato de tafenoquina, WR 238605, WR-238605, Krintafel, N (4) - (2,6-dimetoxi-4- metil-5 - ((3-trifluorometil) fenoxi) -8-quinolinil) -1,4-pentanodiamina	Aminoquinolona, antimalarico ^14^
Profilaxis (profilaxis preexposicion)	Profilaxis previa a la exposición; profilaxis, preexposición	Un metodo de prevencion de enfermedades que implica la administracion de medicamentos a personas en riesgo que no han estado expuestas al agente causante de la enfermedad. Año de introduccion: 2015 ^15^
Recaida	Recurrencias, recrudecimiento, recaídas	El regreso de un signo, síntoma o enfermedad después de una remisión. Año de introduccion: 1991 ^16^

### Selección de estudios

Tres autores de la revisión examinaron de forma independiente los estudios detectados por la estrategia de búsqueda y obtuvieron informes de artículos que podían someterse a la revisión. Se aplicaron los criterios de inclusión expuestos en la pregunta PICOT y se excluyeron las personas menores de 16 años, embarazadas e infectados o coinfectados por *P. falciparum*, los artículos que no estuvieran en inglés o español y los de tipo observacional descriptivo. En los casos de desacuerdo con los criterios o los artículos incluidos, se recurrió al consenso. Se incluyeron estudios clínicos, clínicos aleatorizados y clínicos controlados, revisiones sistemáticas que incluyeran metaanálisis y estudios observacionales analíticos. Se excluyeron los estudios clínicos observacionales descriptivos.

### Extracción de datos y evaluación de la calidad de los estudios

Se extrajeron los datos de los estudios seleccionados y se registraron los resultados de forma independiente. Se elaboró una matriz en el programa Excel (2016), tomando como guía el “Manual de Revisión Sistemática Cochrane”. Se incluyeron las siguientes características: resumen, revista, autor, año de publicación, tipo de muestra, intervención, medidas de asociación u otras medidas estadísticas y resultados en los que se especificaran las limitaciones. Después de este proceso, se seleccionaron los 16 artículos objeto de la revisión final y se sometieron a la revisión crítica en la matriz Caspe, utilizando la herramienta de análisis de revisiones sistemáticas y estudios clínicos. Los artículos con un puntaje de más del 70 % fueron incluidos ^17^.

### Análisis cuantitativo: análisis de heterogeneidad

Se evaluó con la prueba estadística Q de Cochran, se estableció que la variabilidad entre estudios no existía como hipótesis nula, y se utilizó el I2 para medir el grado de inconsistencia de los resultados de los estudios debido a la heterogeneidad y no al azar, fijando el valor >70 % como gran heterogeneidad. Se sometieron tres artículos a análisis cuantitativo, por lo que no fue posible construir un gráfico de embudo (*funnel plot*) para evaluar el sesgo de publicación, y se calculó el índice de tolerancia de Rosenthal como medida para su análisis.

## Resultados

**Figura 1:**
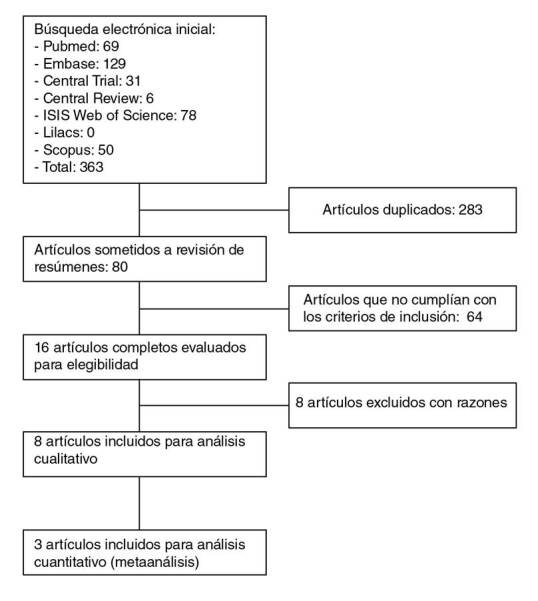
Diagrama de flujo PRISMA del proceso de inclusión y exclusión de estudios

La revisión sistemática incluyó 363 artículos, de los que se excluyeron 283 por presentar resultados repetidos; los resúmenes de 80 artículos se revisaron y quedaron 16 para la lectura crítica utilizando la matriz Caspe. De estos, ocho cumplieron los criterios de inclusión y exclusión establecidos en la pregunta de investigación. El proceso de selección se muestra en el diagrama de flujo PRISMA en la figura 1 y, el resumen de los hallazgos, en los cuadros 2a y 2b, en tanto que el análisis de sesgo se resume en el cuadro 3 y se presenta de forma detallada en el material suplementario con una descripción y el resumen de cada estudio.

**Cuadro 2a:** Resumen de resultados de los estudios incluidos en la evaluación de la calidad en la matriz Caspe

Título	Revista	Lugar	Autor	Año	Tipo de estudio
Randomized, double-blind study of the safety, tolerability, and efficacy of tafenoquine versus mefloquine for malaria prophylaxis in nonimmune subjects	Antimicrob Agents Chemother	Timor Oriental, Australia	Nasveld, *et al.*	2010	Fase III, ensayo de seguridad, tolerabilidad y efectividad de la tafenoquina en la profilaxis de malaria.
Tafenoquine plus chloroquine for the treatment and relapse prevention of Plasmodium vivax malaria (DETECTIVE): A multicentre, double- blind, randomized, phase 2b dose-selection study	The Lancet	Perú, Brasil, Tailandia e India	Llanos-Cuentas, *et al*.	2014	Fase 2b, estudio multicéntrico, doble ciego, aleatorizado, controlado con placebo
Tafenoquine for preventing relapse in people with *Plasmodium vivax* malaria	Cochrane Database Syst Rev	-	Rajapakse, *et al.*	2015	Revisión sistemática y metaanálisis
Estimation of the antirelapse efficacy of tafenoquine using Plasmodium vivax genotyping	J Infect Dis	Perú, Brasil, Tailandia e India	Beck, *et al.*	2016	Fase 2b, estudio multicéntrico, doble ciego, aleatorizado, controlado con placebo
A randomized, double-blind, active-control trial to evaluate the efficacy and safety of a three- day course of tafenoquine monotherapy for the treatment of *Plasmodium vivax* malaria	PLoS ONE	Tailandia	Fukuda, *et al.*	2017	Estudio aleatorizado, de control activo, doble ciego, doble simulación
Tafenoquine for primary and terminal prophylaxis of malaria in apparently healthy people: A systematic review	Trans R Soc Trop Med Hyg	-	Rodrigo, *et al.*	2019	Revisión sistemática y metaanálisis
Tafenoquine versus primaquine to prevent relapse of *Plasmodium vivax* malaria	N Engl J Med	Perú, Brasil, Colombia, Vietnam, y Tailandia	L*lanos-Cuentas, et al.*	2019	Pase 3, contralado, prospectivo, doble ciego, aleatorizado
Single-dose tafenoquine to prevent relapse of Plasmodium vivax malaria	N Engl J Med	Etiopia, Perú, Brasil, Camboya, Tailandia	Lacerda, *et al.*	2019	Multicéntrico, controlado, doble ciego, en paralelo, aleatorizado

**Cuadro 2b:** Resumen de resultados de los estudios incluidos en la evaluación de la calidad en la matriz Caspe

Artículo	Muestra	Intervención	Análisis estadístico	Profilaxis/tratamiento
Nasveld, *et al*., 2010	663/654	1. TQ 200 mg diarios x 3 días seguido de 200 mg semanales x 26 ± 4 semanas 2. MQ 250 mg diarios x 3 días seguido de 250 mg/ semanal x 26 ± 4 semanas Al regreso a Australia los soldados recibían en el grupo de TQ placebo y en el del grupo MQ, el esquema de PQ de 15 mg cada 12 horas x 14 días.	Ji al cuadrado con corrección de Yates o test exacto de Fisher, no se realizaron ajustes para pruebas múltiples. Análisis por protocolo	Profilaxis
Llanos, *et al*., 2014	424/329	1. TQ 50, 100, 300, y 600 mg (dosis única en día 1 o 2) más CQ 600 mg (día 1), 600 mg (día 2), 300 mg (día 3) Vs. placebo 2. PQ 15 mg diarios x 14 días más CQ 600 mg (día 1), 600 mg (día 2), 300 mg (día 3) Vs. Placebo 3.CQ 600 mg (día 1), 600 mg (día 2), 300 mg (día 3) sin otro medicamento	Estimaciones de Kaplan Meier, test de Log Rank Análisis por intención de tratar	Tratamiento
Rajapakse, *et a*l., 2015	5	1. TQ (50 a 100 mg, 300 mg, 500 a 600 mg y 1.800 a 3.000 mg) más CQ 600 mg (día 1), 600 mg (día 2), 300 mg (día 3) Vs. CQ sin otro medicamento. 2. TQ (300 mg única dosis, 600 mg única dosis y 1.800 a 2.100 mg) Vs. PQ (15 mg/día por 14 días)	Riesgo relativo	Tratamiento
Beck, *et al.*, 2016	329/100	1. TQ 50 mg, 100 mg, 300 mg, 600 mg (dosis única en día 1 o 2) más cloroquina 600 mg (día 1), 600 (día 2), 300 (día 3) 2. Primaquina 15 mg diarios x 14 días más cloroquina 600 mg (día1), 600 mg (día 2), 300 mg (día 3) 3. Cloroquina 600 mg (día 1), 600 (día 2), 300 mg (día 3) sin otro medicamento	Estimaciones de Kaplan-Meier, test de Log Rank	Tratamiento
Fukuda, *et al.*, 2017	70/55	1. TQ 400 mg diarios por x 3 días más CQ placebo diario x 3 días más PQ placebo día 4-18 2. TQ placebo diarios por x 3 días más CQ 1.000 mg diarios x 2 días, en el día 3, 500 mg CQ más PQ 15 mg diarios x 14 días	No se planifico una comparación formal entre los grupos de tratamiento, IC (Clopper-Pearson, 90 %). Análisis por protocolo	Tratamiento
Rodrigo, *et al*., 2019	428/6	1. TQ 50, 100, 200 y 400 mg/semana y 400 mg/mes Vs. placebo	Riesgo relativo	Profilaxis
Llanos, *et al.*, 2019	369/251	1. TQ 300 mg (dosis única en día 1 o 2) más CQ 600 mg (día 1), 600 mg (día 2), 300 mg (día 3) 2. PQ 15 mg diarios x 14 días más CQ 600 mg (día 1), 600 mg (día 2), 300 mg (día 3)	Estimaciones de Kaplan-Meier Análisis por protocolo e intención de tratar	Tratamiento
Lacerda*, et al*., 2019	683/522	1. TQ 300 mg dosis única en día 1 o 2 más CQ 600 mg (día 1), 600 mg (día 2), 300 mg (día 3) Vs. placebo 2. PQ 15 mg diarios x 14 días más CQ 600 mg (día 1), 600 mg (día 2), 300 mg (día 3) Vs. placebo	Estimaciones de Kaplan-Meier, modelo de riesgos y proporciones de Cox Análisis por intención de tratar	Tratamiento

TQ: tafenoquina; QC: cloroquina; PQ: primaquina; MQ: mefoloquina

**Cuadro 3:** Resumen del riesgo de sesgo: los juicios de los revisores sobre cada elemento de riesgo de sesgo se encuentran en los anexos

	Sesgo de Realización (cegamiento de los participantes y del personal)	Sesgo de selección (aleatorización)	Sesgo de Detección (cegamiento de los evaluadores del resultado)	Sesgo de desgaste (datos de resultados incompletos)	Sesgo de notificación (notificación selectiva)	Sesgo de publicación (metaanálisis)
Nasveld*, et al.*, 2010	Bajo riesgo	Bajo riesgo	Poco claro	Bajo riesgo	Bajo riesgo	-
Llanos-Cuentas, *et al*., 2014	Bajo riesgo	Bajo riesgo	Bajo riesgo	Bajo riesgo	Bajo riesgo	-
Rajapakse, *et al.,* 2015	-	-	-	-	-	No claro
Beck, *et al.,* 2016	Bajo riesgo	Bajo riesgo	Poco claro	Bajo riesgo	Bajo riesgo	-
Fukuda, *et al.*, 2017	Bajo riesgo	Bajo riesgo	Bajo riesgo	Bajo riesgo	Bajo riesgo	-
Rodrigo, *et al.*, 2019	-	-	-	-	-	No claro
Llanos-Cuentas, *et al*., 2019	Bajo riesgo	Bajo riesgo	Poco claro	Bajo riesgo	Bajo riesgo	-
Lacerda, *et al*., 2019	Bajo riesgo	Bajo riesgo	Poco claro	Bajo riesgo	Bajo riesgo	-

Se analizó en dos etapas la historia natural de la enfermedad, estableciendo como profilaxis la intervención en personas sanas que hubieran estado expuestas y, como tratamiento, la intervención en personas con diagnóstico microbiológico de malaria por *P. vivax*. En esta definición se incluyó la cura radical del hipnozoíto a los seis meses como factor determinante para evaluar la eficacia.

### Eficacia de la tafenoquina en la profilaxis de la malaria por Plasmodium vivax

*Tafenoquina versus mefloquina.* En el 2010, Nasveld, *et al*. ^18^, realizaron un estudio de fase III sobre seguridad, tolerabilidad y efectividad de los medicamentos en soldados australianos que iban a realizar una misión en la isla de Timor. Se comparó la administración de 600 mg de tafenoquina (200 mg/día durante tres días) seguidos de 200 mg por semana durante 26 ± 4 semanas con la de 750 mg de mefloquina (250 mg/día por tres días), seguidos de 250 mg semanales por el mismo periodo. Se hizo el seguimiento microbiológico en las semanas 4, 8, 16 y 26, y en los dos grupos hubo reportes en que se descartó la presencia de la enfermedad. Se llevó a cabo un análisis por protocolo, con resultados que evidenciaron cuatro casos (0,9 %) de infección por *P. vivax* en el grupo de tafenoquina y un solo caso (0,7 %) en el grupo de mefloquina durante la fase de seguimiento de la recaída en las semanas 16 y 20 (IC_95%_ -1,32 a 1,74; p=1,0).

En el 2019, Rodrigo, *et al.*^19^, hicieron una revisión sistemática con metaanálisis en la que incluyeron dos artículos: el primero comparaba la tafenoquina en dosis que variaban de 25 a 200 mg semanales con la mefloquina en dosis de 250 mg semanales durante 12 semanas, en tanto que, en el otro, se comparaba la administración de 200 mg semanales de tafenoquina con 250 mg semanales de mefloquina durante 26 semanas. En el análisis cuantitativo, se reportó una heterogeneidad del 0 %, con un riesgo relativo (RR) de 1,0 (IC_95%_ 0,45-2,24), efecto nulo, y ausencia de significación estadística. Ninguno de los dos estudios reportó casos positivos para *P. vivax*.

*Tafenoquina versus placebo.* Se encontró solo una revisión sistemática en que se sometieron a análisis cuantitativo tres artículos de Ghana, Kenia y Tailandia, con un rango de dosis desde los 25 mg hasta los 400 mg semanales o mensuales. Se reportó un efecto total a favor de la tafenoquina (RR=0,12; IC_95%_ 0,09-0,16). En dos de los estudios, se estandarizó la dosis de tafenoquina (200 mg), lo que mostró un resultado también favorable en comparación con el placebo (RR=0,14; IC_95%_ 0,09-0,21) (19)MEDLINE [PubMed], Embase [Ovid], Scopus, CINAHL [EBSCOhost] and LILACS.

### Eficacia de la tafenoquina en el tratamiento de la malaria por *Plasmodium vivax*

En tres estudios clínicos aleatorizados, se evaluó la eficacia en las etapas aguda y latente a los seis meses, con un análisis por intención de tratar con base en estimaciones mediante la función de supervivencia y el método de Kaplan-Meier.

Llanos-Cuentas, *et al*. (2014), evaluaron la eficacia de la tafenoquina con relación a la ausencia de recaída a los 6 meses y obtuvieron mejores resultados en los dos grupos con mayores dosis; en el grupo de 300 mg se obtuvo una eficacia del 89,2 % (IC_95%_ 77-95) y, en el grupo de 600 mg, fue de 91,9 % (IC_95%_ 80-97) ^12^. En comparación con la cloroquina sola, la eficacia fue significativamente mayor al agregar los 300 mg de tafenoquina (diferencia de tratamiento: 51,7 %; IC_95%_ 35-69; p<0,0001). En un análisis *post hoc* solicitado por *The Lancet*, se comparó el esquema terapéutico de tafenoquina en dosis de 300 mg y la cloroquina sin adición de otro medicamento. Se obtuvo un *odds ratio* (OR) de 8,4 (IC_95%_ 3,4-20,6) para la eficacia en ausencia de recaídas a los 6 meses.

Llanos-Cuentas, *et al*. (2019), también evaluaron en un estudio clínico de fase III, la eficacia en la ausencia de recaída a los 6 meses de dos esquemas de tratamiento comparados con placebo; en el grupo de tafenoquina en dosis única de 300 mg comparada con la cloroquina la eficacia fue de 73,3 % (IC_95%_ 64,8-79,2) y en el grupo de primaquina con cloroquina fue de 75,1 % (IC_95%_ 64,2-83,2) ^10^.

Lacerda, *et al.* (2019), en un estudio multicéntrico realizado en Etiopia, Perú, Brasil, Camboya y Filipinas, evaluaron el mismo resultado y tipo de intervención del estudio previo. La razón de riesgo (HR) para el riesgo de recurrencia fue de 0,30 (IC_95%_ 0,22-0,40) en el grupo de la tafenoquina, comparada con un placebo (p<0,001), y de 0,26 (IC95%: 0,18 - 0,39), en el grupo de primaquina comparada con un placebo (p<0,001) ^20^.

En esta revisión, solo se encontró un análisis por protocolo realizado por Llanos-Cuentas, *et al*. (2019), ^10^. Fukuda, *et al*. (2017), realizaron un estudio en Tailandia y encontraron que en el grupo de la tafenoquina no se presentaron recaídas a los 6 meses, en tanto que, en el de primaquina- cloroquina, en el día 63 un paciente presentó este resultado. No se reportaron datos estadísticos ^21^.

Rajapakse, *et al*. (2015), llevaron a cabo una revisión sistemática con análisis cuantitativo de dos esquemas de tratamiento: en el primero se comparaba la eficacia a los 6 meses de la tafenoquina con cloroquina y de la cloroquina sola, sin otro medicamento. Se reportó una heterogeneidad del 31 % y un RR de 0,13 (IC_95%_ 0,08-0,22). El segundo tratamiento combinaba tafenoquina con cloroquina y se comparó con el esquema de primaquina: se registró únicamente un estudio en que se usó una dosis de tafenoquina de 300 mg y el RR fue de 0,41 (IC_95%_ 0,15-1,14) y otro con tafenoquina en dosis de 1.800 a 2.100 mg con un RR de 0,06 (IC_95%_ 0,15-0,59). En el resultado final, hubo una heterogeneidad de 0 % (RR=0,3; IC_95%_ 0,15-0,59) a favor de la tafenoquina ^22^.

A partir de la muestra que utilizaron Llanos-Cuentas, *et al*. (2014) ^12^, Beck, *et al*. (2016), ^23^ hicieron un estudio de la eficacia de la tafenoquina según la clasificación genotípica del parásito. Para esto, se agruparon los alelos por tamaño de bandas para las regiones codificantes y por longitud de repetición de los microsatélites (3 pares de bases para MS16 y 4 pares de bases para Pv3.27). Si las recaídas mostraban al menos un marcador diferente al de la infección inicial, se clasificaban como heterogéneas, pero si había coincidencia de la secuencia genómica, se consideraban homólogas. Se utilizaron estimaciones de Kaplan-Meier y en las secuencias heterólogas no se encontró significación estadística para las dosis altas y bajas de tafenoquina, con el 6,7 y el 15,2 %, respectivamente, y una diferencia de -8,5 % (IC_95%_ −17,9-1,0; p=0,069). En las secuencias homólogas se evidenció una recurrencia con relación dosis-respuesta: 2,9 % para las dosis altas de tafenoquina comparado con el 31,2 % con las dosis bajas, y una diferencia de tratamiento de -28,3 % (IC_95%_ −37,9% a −18,7%; p<0,001) ^23^. No se encontraron estudios adicionales que utilizaran la diferenciación genotípica.

## Metaanálisis de la diferencia de riesgo de los esquemas de tafenoquina y de primaquina

**Figura 2:**
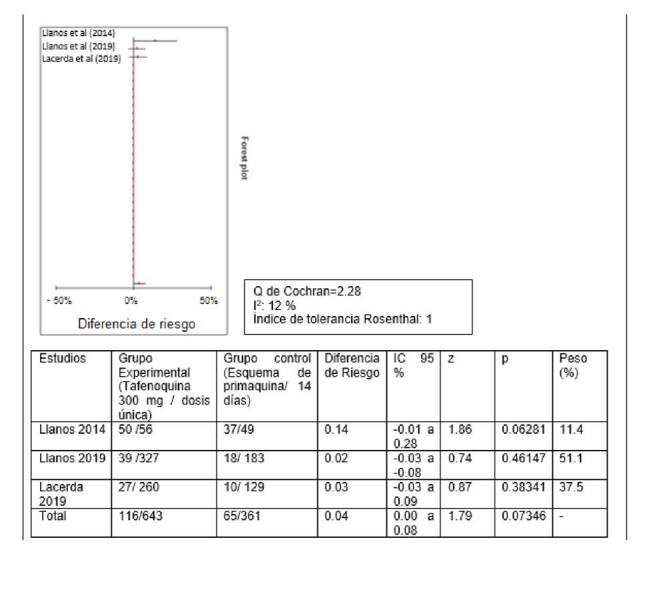
Diagrama de bosque de la diferencia de riesgo (tafenoquina*Vs. primaquina**) a los 6 meses para recaída de infección por *Plasmodium vivax*

Se hizo el análisis cuantitativo de tres estudios con análisis por intención de tratar: el de Llanos-Cuentas, *et al*. (2014) ^12^, el de Llanos-Cuentas, *et al*. (2019) ^10^, y el de Lacerda, *et al*. (2019) ^20^. En los tres se comparaba el esquema de tafenoquina con dosis única de 300 mg en el día 1 o el 2 en conjunto con la cloroquina en dosis de 600 mg en el día 1, de 600 mg en el día 2, y de 300 mg en el día 3; y el esquema de primaquina en dosis de 15 mg/día durante 14 días en conjunto con cloroquina en dosis de 600 mg en el día 1, de 600 mg en el día 2, y de 300 mg en el día 3. Se obtuvo un bajo nivel de heterogeneidad, por lo que se utilizó un modelo de efectos fijos para estimar el efecto combinado de la diferencia de riesgos con el método de Mantel-Haenszel.La diferencia de riesgo total fue de 0,04 (IC_95%_ 0 - 0,08; p=0,073); se aceptó la hipótesis nula de que no había diferencia en la eficacia de los dos esquemas para evaluar la recaída a los 6 meses (figura 2).

## Discusión

La tafenoquina en dosis mayores de 25 mg como medida de profilaxis puede ser mejor que el placebo, pero no demostró superioridad sobre la mefloquina durante el periodo estudiado. Rodrigo, *et al*. (2019) ^19^, coinciden con este hallazgo.

Es importante determinar el tipo de análisis en el tratamiento: el esquema de tafenoquina en una única dosis conjuntamente con cloroquina ha sido un avance en cuanto al cumplimiento, por lo que un análisis por protocolo permitiría inferir la eficacia, disminuyendo el sesgo de seguimiento. Sin embargo, el análisis por intención de tratar es necesario, ya que los grupos de comparación de los estudios incluidos presentan un esquema de tratamiento de 14 días. Los autores controlaron este sesgo mediante un seguimiento clínico periódico, asegurando al máximo el cumplimiento a los seis meses del estudio. Llanos-Cuentas, *et al*. (2019) ^10^, incluyeron estos dos análisis con resultados similares.

En todos los estudios se estableció el esquema de tafenoquina más cloroquina como tratamiento en la fase aguda y la latente, con dosis de 300 mg de tafenoquina y 600 mg de cloroquina. Este esquema mostró una mayor eficacia en comparación con el esquema de primaquina más cloroquina y el de cloroquina sola. Únicamente un estudio multicéntrico, doble ciego, en paralelo, aleatorizado y controlado con placebo, el de Lacerda, *et al*. (2019), evidenció una menor eficacia en el análisis por intención de tratar de la tafenoquina frente a la primaquina. Hace falta más evidencia para determinar si el esquema con primaquina es superior al de tafenoquina. En los artículos analizados, se evaluó la eficacia en el tratamiento de la tafenoquina, utilizando como medida la ausencia de recaída a los 6 meses, pero hacen falta estudios con un periodo de seguimiento más largo para determinar la cura radical de la malaria por *P. vivax*.

El análisis cuantitativo con bajo riesgo de heterogeneidad evidenció que no hubo diferencia en la eficacia del esquema de tafenoquina más primaquina, por lo que, al no ser inferior la primera y presentar la ventaja de administrarse en una única dosis, podría mejorar el cumplimiento del tratamiento y la cura de la infección por *P. vivax* tanto en la fase aguda como en la latente.

Una limitación del presente estudio fue el reducido número de artículos que cumplían los criterios de inclusión como consecuencia de la poca evidencia disponible, razón por la que el índice de tolerancia de Rosenthal fue bajo.

Se encontró una revisión sistemática con metaanálisis y búsqueda activa hasta el 13 de abril de 2015, con criterios de inclusión similares (Rajapakse, *et al*., 2019) ^22^. Sin embargo, nuestro estudio establece un periodo de 10 años (2009-2019) e incluye a la población con déficit de G6PD como factor de riesgo para presentar hemólisis secundaria a la administración de 8-aminoquinoleínas, pero en la estrategia de búsqueda no se encontraron artículos que hubieran condiderado esta enfermedad recesiva ligada al cromosoma X como criterio de inclusión, por lo que los resultados no pueden ser aplicados en esta población.

Aunque aún hace falta evidencia, Rueangweerayut, *et al*. (2017) ^24^, iniciaron estudios clínicos de cohorte abierta con relación dosis-respuesta para evaluar la seguridad de la tafenoquina en sujetos heterocigotos con rango de actividad enzimática de G6PD del 40 al 60 %, en la cual reportaron que la tafenoquina en dosis única de 300 mg no aumentó la gravedad de la hemólisis en comparación con la primaquina en dosis de 15 mg/día durante 14 días.

La prevalencia calculada por la OMS en Colombia para la deficiencia de la G6PD es del 3 al 7 %. Los estudios poblacionales son escasos. En el 2008, se hizo uno transversal para medir la prevalencia de esta enfermedad en la población masculina sana de Turbo, Antioquia, y en la población con diagnóstico de *P. vivax*. Los resultados indicaron que el 14,8 % de la muestra tuvo déficit de G6PD y, entre las personas que presentaban malaria por *P. vivax,* el 9,5 % presentaba esta enfermedad ^25^^,^^26^.

La tafenoquina es un medicamento aprobado recientemente por organizaciones sanitarias como la FDA y la TGA, que ha mostrado eficacia en el tratamiento de la infección por *P. vivax,* pero se requieren estudios de costo- efectividad que contemplen el déficit de G6PD para poderla utilizar en esta población de riesgo. Esta nueva alternativa de tratamiento es una opción para mejorar el control de la enfermedad en Colombia y acercarse a las metas planteadas en la Estrategia técnica mundial contra la malaria 2016-2030*.*

Al igual que la primaquina, la tafenoquina previene la recaída de malaria por *P. vivax,* por lo que la evidencia revisada permite concluir que puede recomendarse para su tratamiento. Es importante mencionar que el déficit de G6PD es un error innato del metabolismo que podría someterse a tamizaje neonatal o a detección mediante una prueba previa al tratamiento con 8-aminoquinoleínas en personas en alto riesgo de paludismo, lo que facilitaría la vigilancia del tratamiento, en especial con la tafenoquina, que, aunque mejora el cumplimiento por administrarse en una única dosis, tiene una vida media más prolongada y se encuentra en estudio para determinar su potencial hemolítico. El costo del tamizaje varía de acuerdo con la técnica y oscila entre COP $ 100.000 y COP $ 1’000.000.

En este estudio se encontró que el esquema de tafenoquina, en dosis única de 300 mg en el día 1 o el día 2, en conjunto con cloroquina, en dosis de 600 mg en el día 1, 600 mg en el día 2 y 300 mg en el día 3, y el esquema de primaquina, 15 mg por día por 14 días, en conjunto con cloroquina, 600 mg en el día 1, 600 mg en el día 2 y 300 mg en el día 3, muestran que la diferencia de riesgo total fue de 0,04 (IC_95%_ 0 - 0,08; p=0,073), lo cual indica que no hay diferencia en la eficacia de los dos esquemas para evaluar la recaída a los seis meses. Sin embargo, al ser el de tafenoquina un tratamiento de corta duración, sí implica una mejora significativa en el cumplimiento del tratamiento.

Todos los estudios incluidos evaluaron las recurrencias como una medida indirecta de la recaída, ya que los participantes permanecieron en el área endémica durante el seguimiento, con riesgo de reinfección. Llanos-Cuentas, *et al*. (2014) ^12^, mostraron que la tafenoquina en dosis bajas únicas (50 y 100 mg) fue ineficaz para prevenir las recurrencias, en comparación con la monoterapia con cloroquina, y que 300 mg pueden ser más adecuados para esta indicación. Lacerda, *et al*. (2019) ^20^, probaron esta hipótesis con un tamaño de muestra mayor y la confirmaron.

La OMS ya recomienda una dosis de primaquina superior a la estándar en Asia oriental y Oceanía (30 mg/día durante 14 días) y es posible que se requiera la misma para la tafenoquina, además de revisar su eficacia y seguridad ^27^.

La hemólisis con deficiencia de G6PD es un riesgo estructuralmente similar con la primaquina y la tafenoquina, y se aplican las mismas precauciones en su prescripción. La OMS ha mencionado que, cuando la actividad enzimática es inferior al 30 % de lo normal, se puede prescribir un esquema modificado (0,75 mg base/kg/día/semana durante 8 semanas) bajo supervisión médica estricta tanto para hombres como para mujeres no embarazadas ^25^^,^^26^.

En este contexto, es posible que en futuros estudios experimentales se comparen la tafenoquina y la primaquina con esquemas de dosificación modificados en estas circunstancias. Las observaciones de los estudios incluidos demuestran que, con una dosis única de 300 mg, es probable que la tafenoquina no cause metahemoglobinemia sintomática.

Al considerar el panorama general, se concluye que la tafenoquina es más eficaz que el placebo para prevenir las recaídas de malaria por *P. vivax* durante un seguimiento de seis meses a partir de evidencia de certeza moderada. Sin embargo, las recaídas de la malaria por *P. vivax* pueden ocurrir incluso más tarde, probablemente hasta un año después, situación que amerita investigaciones futuras.

El tipo más común de efecto adverso grave informado fue una disminución de la concentración de hemoglobina, por lo que los médicos deben estar alerta para hacer seguimiento a los pacientes recetados que tienen un valor bajo de hemoglobina inicial.

En su metaanálisis de estudios con participante individual por protocolo, Lacerda, *et al*. (2019) ^20^, y Llanos-Cuentas, *et al*. (2019) ^10^, llegaron a la misma conclusión que nosotros, aunque no se pudo demostrar que la tafenoquina no fuera inferior a la primaquina con base en un margen de no inferioridad preestablecido calculado a partir del resultado de Llanos-Cuentas, *et al*. (2014) ^12^.

En cuanto a posibles sesgos en el proceso de revisión, se buscaron en los registros de estudios mediante estrategias de búsqueda específicas para detectar los no publicados y no se encontró ninguno.

## Archivos suplementarios

**Características de los estudios**

**Table:**

Artículo	Randomized, double-blind study of the safety, tolerability, and efficacy of tafenoquine versus mefloquine for malaria prophylaxis in nonimmune subjects Nasvelt, *et al*., (2010)
Autores	Peter E. Nasveld, Michael D. Edstein, Mark Reid, Leonard Brennan, Ivor E. Harris, Scott J. Kitchener, Peter A. Leggat, Philip Pickford, Caron Kerr, Colin Ohrt, William Prescott, and the Tafenoquine Study Team
Criterios de selección:	Inclusión: sujetos masculinos y femeninos que tenían entre 18 y 55 años de edad, considerados saludable por antecedentes médicos y examen físico con valores hematológicos y bioquímicos normales, G6PD normal, estar dispuesto y ser capaz de dar su consentimiento informado por escrito y cumplir con el protocolo del estudio Exclusión: las mujeres fueron excluidas si estaban embarazadas, lactando o no querían utilizar métodos anticonceptivos reconocidos.
Resultados	Eficacia de la profilaxis a 6 meses y eficacia del tratamineto anti-recaída en personas sanas con exposicion a 6 meses
Sesgo	Sesgo de realización: estudio doble ciego (bajo) Sesgo de selección: un sistema de aleatorización en bloques para una proporción de 3:1 (bajo) Sesgo de detección: el cegamiento de los evaluadores no está determinado. (poco claro) Sesgo de notificación: se dispone del protocolo de estudio y todos los resultados de interés de la revisión están descritos de la forma prevista en el protocolo (bajo) Sesgo de desgaste: la proporción de datos faltantes o su posible efecto no es suficiente para tener un impacto clínicamente relevante (bajo)

**Table:**

Artículo	Tafenoquine plus chloroquine for the treatment and relapse prevention of *Plasmodium vivax* malaria (DETECTIVE): A multicentre, double-blind, randomised, phase 2b dose-selection study Llanos, *et al*., (2014)
Autores	Alejandro Llanos-Cuentas, Marcus V Lacerda, Ronnatrai Rueangweerayut, Srivicha Krudsood, Sandeep K Gupta, Sanjay K Kochar, Preetam Arthur, Nuttagarn Chuenchom, Jörg J Möhrle, Stephan Duparc, Cletus Ugwuegbulam, Jörg-Peter Kleim, Nick Carter, Justin A Green, Lynda Kellam
Criterios de selección:	*P. vivax* no complicado confirmado microscópicamente, monoinfección selección con una densidad parasitaria asexual de más de 100 por μl de sangre y menos de 100.000 por μl sangre Exclusión: tratamiento antipalúdico en los últimos 30 días, paludismo grave, vómito intensos, hemoglobina de menos de 70 g /L, mujeres embarazadas o lactantes, paciente con actividad enzimática G6PD <70%, pacientes femeninas con actividad enzimática G6PD <90% ingresaban solo si tenían hemoglobina de 100 g/L o más.
Resultados	No se hizo ninguna distinción entre la recurrencia de P. vivax causada por recrudescencia, reinfección o recaída por activación de hipnozoitos. El resultado principal fue la eficacia de pacientes que estaban libre de recaídas a los 6 meses.
Sesgo	Sesgo de realización: doble ciego (bajo) Sesgo de selección: una asignación al azar generada por un programa de forma estratificado por el recuento de parásitos de línea de base (≤7.500 y >7.500 por μl de sangre) (bajo) Sesgo de detección: el cegamiento de los evaluadores es correcto y es poco probable que se haya roto (bajo) Sesgo de notificación: se dispone del protocolo de estudio y todos los resultados de interés de la revisión están descritos de la forma prevista en el protocolo (bajo) Sesgo de desgaste: la proporción de datos faltantes o su posible efecto no es suficiente para tener un impacto clínicamente relevante (bajo)

**Table:**

Artículo	Tafenoquine for preventing relapse in people with *plasmodium vivax* malaria (Review) Rajapakse,2015
Autores	Rajapakse S, Rodrigo C, Fernando SD
Criterios de selección:	Inclusión: a dultos y niños con diagnóstico confirmado (clínico y parasitológico) de paludismo por *P. vivax* Exclusión: estudios en que las personas con deficiencia de G6PD se han excluido y en poblaciones sin detección de deficiencia de G6PD, estudios aleatorizados sobre profilaxis, estudios no controlados, informes de casos y estudios farmacocinéticos
Resultados	Parasitemia recurrente por *P. vivax* durante seis meses de seguimiento
Sesgo	Sesgo de publicación: no evaluado (poco claro)

**Table:**

Artículo	Estimation of the antirelapse efficacy of Tafenoquine, using *Plasmodium vivax* genotyping Beck,*et al*. (2016)
Autores	Hans Peter Beck, Rahel Wampfler, Nick Carter, Gavin Koh, Lyda Osorio, Ronnatrai Rueangweerayut, Srivcha Krudsood, Marcus V. Lacerda, Alejandro Llanos Cuentas, Stephan Duparc, Justin P. Rubio y Justin A. Green
Criterios de selección:	Inclusión: *P. vivax* no complicado confirmado microscópicamente, monoinfección con una densidad parasitaria asexual de más de 100 por μl de sangre y menos de 100.000 por μl sangre Exclusión: tratamiento antipalúdico en los últimos 30 días, paludismo grave, vómito intensos, hemoglobina de menos de 70 g/L, mujeres embarazadas o lactantes, paciente con actividad enzimática G6PD <70 %, pacientes femeninas con actividad enzimática G6PD <90 % ingresaban solo si tenían hemoglobina de 100 g/L o más.
Resultados	- Recurrencia homóloga de *Plasmodium vivax* a 6 meses - Recurrencia heteróloga de *Plasmodium vivax* a 6 meses
Sesgo	Sesgo de realización: doble ciego (bajo) Sesgo de selección: aleatorizado (bajo) Sesgo de detección: no se menciona cómo se realizó no se menciona cómo se realizó el cegamiento de los evaluadores (poco claro) Sesgo de notificación: se dispone del protocolo de estudio y todos los resultados de interés de la revisión están descritos de la forma prevista en el protocolo (bajo) Sesgo de desgaste: la proporción de datos faltantes o su posible efecto no es suficiente para tener un impacto clínicamente relevante (bajo)

**Table:**

Artículo	A randomized, double blind, active control trial to evaluate the efficacy and safety of a three day course of tafenoquine monotherapy for the treatment of *Plasmodium vivax* malaria Fukuda, *et al*. (2016)
Autores	Mark M. Fukuda, Srivicha Krudsood, Khadeeja Mohamed, Justin A Green, Sukhuma Warrasak, Harald Noedl, Ataya Euswas, Mali Ittiverakul, Nillawan Buathong, Sabaithip Sriwichai, R. Scott Miller, Colin Oh
Criterios de selección:	Inclusión: pacientes masculinos o femeninos de 20 a 60 años con infección por P. selección vivax confirmada microscópicamente en frotis de sangre (densidad de parásitos >500 y <200.000/μl) Exclusión: premenarquia, embarazada o lactando, no deseo de planificar, infección mixta, vómitos graves, o problemas de absorción intestinal, enfermedad renal, cirugía ocular previa, anomalías corneales o retinianas, glaucoma, personas alérgicas a las 8 aminoquinoleínas y los que utilizaron antimaláricos en los últimos 30 días
Resultados	- Eficacia en el día 28, definida parasotológicamente - Proporción de pacientes sin reaparición de parasitemia por P. vivax en los días 60, 90 y 120
Sesgo	Sesgo de realización: doble ciego (bajo) Sesgo de selección: aleatorizado (bajo) Sesgo de detección: el cegamiento de los evaluadores es correcto y es poco probable que se haya roto (bao) Sesgo de notificación: se dispone del protocolo de estudio y todos los resultados de interés de la revisión están descritos de la forma prevista en el protocolo (bajo) Sesgo de desgaste: la proporción de datos faltantes o su posible efecto no es suficiente para tener un impacto clínicamente relevante (bajo)

**Table:**

Artículo	Tafenoquine for primary and terminal prophylaxis of malaria in apparently healthy people: A systematic review Rodrigo, *et al.* (2019)
Autores	Chaturaka Rodrigo, Senaka Rajapakseb y Sumadhya Deepika Fernando
Criterios de selección:	Inclusión: todos los diseños de estudios clínicos, incluyendo estudios abiertos que han utilizado tafenoquina como tratamiento en profilaxis primaria o terminal en adultos y niños sanos al inicio del estudio Exclusión: estudios que inscribieron a pacientes con diagnóstico de paludismo sintomático o asintomático al inicio del estudio
Resultados	Eficacia de la profilaxis a 6 meses y eficacia del tratamiento anti recaída en personas sanas con exposicion a 6 meses
Sesgo	Sesgo de publicación: no evaluado (poco claro)

**Table:**

Artículo	Tafenoquine versus Primaquine to prevent relapse of *Plasmodium vivax* malaria Llanos, *et al.* (2019)
Autores	A. Llanos Cuentas, M.V.G. Lacerda, T.T. Hien, I.D. Vélez, C. Namaik larp,C.S. Chu, M.F. Villegas, F. Val, W.M. Monteiro, M.A.M. Brito, M.R.F. Costa,R. Chuquiyauri, M. Casapía, C.H. Nguyen, S. Aruachan, R. Papwijitsil, F.H. Nosten, G. Bancone, B. Angus, S. Duparc, G. Craig, V.M. Rousell, S.W. Jones, E. Hardaker, D.D. Clover, L. Kendall, K. Mohamed, G.C.K.W. Koh,V.M. Wilches, J.J. Breton, and J.A. Green
Criterios de selección:	Inclusión: = Llanos (2014) excepto que se incluían las personas con actividad enzimática G6PD >40 % en mujeres y en hombres >70 %. Los pacientes debían tener un nivel de hemoglobina >7 g/dl, excepto las pacientes con actividad moderada de la enzima G6PD, que debían tener un nivel de hemoglobina >8 g/dl Exclusión: = Llanos (2014)
Resultados	Eficacia de la profilaxis a 6 meses y eficacia del tratamineto anti recaída en personas sanas con exposicion a 6 meses
Sesgo	Sesgo de realización: estudio doble ciego (bao) Sesgo de selección: aeatorizado (bajo) Sesgo de detección: el cegamiento de los evaluadores no esta determinado (poco claro) Sesgo de notificación: se dispone del protocolo de estudio y todos los resultados de interés de la revisión están descritos de la forma prevista en el protocolo (bajo) Sesgo de desgaste: la proporción de datos faltantes o su posible efecto no es suficiente para tener un impacto clínicamente relevante (bajo)

**Table:**

Artículo	Single dose Tafenoquine to prevent relapse of *Plasmodium vivax* malaria Lacerda*,et al.*(2019)
Autores	M.V.G. Lacerda, A. Llanos Cuentas, S. Krudsood, C. Lon, D.L. Saunders, R. Mohammed, D. Yilma,D. Batista Pereira, F.E.J. Espino, R.Z. Mia, R. Chuquiyauri, F. Val, M. Casapía, W.M. Monteiro, M.A.M. Brito,M.R.F. Costa, N. Buathong, H. Noedl, E. Diro, S. Getie, K.M. Wubie, A. Abdissa, A. Zeynudin, C. Abebe, M.S. Tada, F. Brand, H. P. Beck, B. Angus, S. Duparc, J. P. Kleim, L.M. Kellam, V.M. Rousell, S.W. Jones, E. Hardaker,K. Mohamed, D.D. Clover, K. Fletcher, J.J. Breton, C.O. Ugwuegbulam, J.A. Green, and G.C.K.W. Koh
Criterios de selección:	Inclusión: >16 años de edad, (≥18 años de edad si está en Etiopía) e infección por P. vivax confirmada microscópicamente (>100 a <100.000 parásitos asexuales por microlitro) y un intervalo QT corregido según fórmula de Fridericia (QTcF) <de 450 mseg. Las participantes tenían pruebas de embarazo negativas, no estaban amamantando y usaban anticonceptivos aprobados Exclusión: infección mixta o paludismo grave, vómito intenso, hemoglobina <7 g/dl, pruebas hepáticas normales, uso de un fármaco antipalúdico en los 30 días previos o participación en estudio previo, alergias a 8 aminoquinoleínas o cloroquina. Pacientes con deficiencia de G6PD
Resultados	El resultado primario de eficacia fue el porcentaje de pacientes que estaban libres de recurrencia a los 6 meses.
Sesgo	Sesgo de realización: estudio doble ciego (bajo) Sesgo de selección: aleatorizado (bajo) Sesgo de detección: el cegamiento de los evaluadores no está determinado. (poco claro) Sesgo de notificación: se dispone del protocolo de estudio y todos los resultados de interés de la revisión están descritos de la forma prevista en el protocolo. (bajo) Sesgo de desgaste: la proporción de datos faltantes o su posible efecto no es suficiente para tener un impacto clínicamente relevante. (bajo)

**Estudios excluidos por la matriz Caspe**

**Table:**

Artículo	Autor	Motivo
Tafenoquine at therapeutic concentrations does not prolong Fridericia-corrected QT interval in healthy subjects	Green, *et al.*	Habla de seguridad, no de eficacia.
Tafenoquine treatment of *Plasmodium vivax* malaria: suggestive evidence that CYP2D6 reduced metabolism is not associated with relapse in the Phase 2b DETECTIVE trial	Jean, *et al.*	Estudio in vitro
Exposure-response analyses for Tafenoquine after administration to patients with *Plasmodium vivax* malaria	Tenero, *et al.*	No habla de eficacia, habla de farmacocinética según la dosis.
Prophylactic efficacy of primaquine for preventing Plasmodium falciparum and *Plasmodium vivax* parasitaemia in travelers: A meta-analysis and systematic review	Kolifarhood, *et al.*	No habla de tafenoquina, solo habla de primaquina.
Comparative ophthalmic assessment of patients receiving tafenoquine or chloroquine/primaquine in a randomized clinical trial for *Plasmodium vivax* malaria radical cure	Warrasak, *et al.*	Solo habla de seguridad oftálmica.
A retrospective analysis of the protective efficacy of tafenoquine and mefloquine as prophylactic anti-malarials in non-immune individuals during deployment to a malaria-endemic area	Dow, *et al.*	Estudio restrospectivo, no se establecen medidas de asociación.
Short-course primaquine for the radical cure of *Plasmodium vivax* malaria: A multicenter, randomized, placebo-controlled non-inferiority trial	Taylor, *et al.*	Solo habla de primaquine.
Hemolytic potential of Tafenoquine in female volunteers heterozygous for glucose-6-phosphate dehydrogenase (G6PD) deficiency (G6PD Mahidol Variant) versus G6PD-normal volunteers	Rueangweerayut, *et al.*	Habla de seguridad, no de eficacia.
